# Expert consensus on clinical trials of human xenotransplantation in China

**DOI:** 10.1002/hcs2.6

**Published:** 2022-07-27

**Authors:** Jiefu Huang, China Organ Transplantation Development Foundation

**Affiliations:** ^1^ China Organ Transplantation Development Foundation Beijing China

**Keywords:** expert consensus, medical ethics, xenotransplantation

## Abstract

The history of xenotransplantation started in the 19th century. After a few decades of investigation, significant breakthroughs and preclinical milestones have been achieved worldwide. With the recent transplantation of genetically modified porcine kidneys and heart into humans, these ground‐breaking achievements have attracted great attention worldwide, in the hope that xenotransplantation might alleviate or even solve the problem of organ shortage. On January 20, 2022, the China Organ Transplantation Development Foundation convened a symposium on “The History, Current Situation and Future of Human Xenotransplantation Clinical Trials,” where ways to promote the ethical and sustainable development of xenotransplantation in China were discussed among the participating experts. A formal consensus was reached as the product of the symposium, outlining the expert opinions on scientific, regulatory, and ethical issues of clinical trials of xenotransplantation in China.

## Graphical abstract

Challenges, milestone, and future priority of xenotransplantation in China.

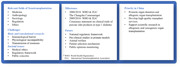

**Abbreviations**
COTDF: China Organ Transplantation Development Foundation
COTRS: China Organ Transplant Response System
IXA: International Xenotransplantation Association
NHC: National Health Commission
PERV: porcine endogenous retrovirus
WHO: World Health Organization

The history of xenotransplantation started from the experiments of skin grafts and corneal transplantation in the 19th century, while the development of solid organ transplantation from animals to humans was not seen until the 1960s [1]. The experiments of kidney, heart, and liver xenotransplantation were attempted without lasting success [2]. After a few decades of investigation, significant breakthroughs and preclinical milestones have been achieved worldwide [3, 4, 5], yet biological barriers and ethical issues have long been preventing the clinical trial of xenotransplantation from being carried out, therefore delaying the clinical application of xenotransplantation in the foreseeable future [6, 7].

In September 2020, at the University of Alabama at Birmingham, two genetically modified porcine kidneys were transplanted to a human brain‐dead decedent [8]. Two‐month later, another similar investigational xenotransplant surgery procedure was performed at the New York University, using the genetically engineered kidneys from a pig without alpha‐gal gene, which is responsible for a rapid antibody‐mediated rejection of porcine organs by humans [9]. In January 2022, with the emergency authorization under the compassionate criteria, a historic first clinical trial of xenotransplantation was carried out at the University of Maryland Medical Centre, with a porcine heart with 10 genetic modifications transplanted into an adult human suffered from end‐stage heart disease [10]. These ground‐breaking achievements have attracted great attention worldwide, in the hope that xenotransplantation might alleviate or even solve the problem of organ shortage.

On January 20, 2022, the China Organ Transplantation Development Foundation (COTDF) convened a symposium on “The History, Current Situation and Future of Human Xenotransplantation Clinical Trials” under the guidance of the Bureau of Medical Administration of the National Health Commission (NHC) of the People's Republic of China. More than 200 participants from NHC, COTDF, China Organ Transplant Response System (COTRS) Scientific Committee, and experts in related fields from hospitals, universities, and research institutions attended the meeting and discussed ways to promote the ethical and sustainable development of xenotransplantation in China. A formal consensus on clinical trials of xenotransplantation was reached as the product of the symposium.

a. Xenotransplantation is an important component of organ transplantation. The scientific research in this branch can further promote the development of organ transplantation technologies. However, a series of challenges must be addressed before the rolling out of clinical trials on humans, including proper evaluation of graft function [11], immunological barrier [12], physiological incompatibility [13], transmission of zoonoses [14], and medical ethics [15]. To tackle some of these issues, innovations have made it possible to use novel immunosuppressant such as the monoclonal antibody against CD40 [4], to select animals with low virus expression rate and then produce source pigs for reduced PERVs expression by means such as small‐interfering RNA [16, 17, 18], or to directly inactivate copies of PERVs by CRISPR/Cas9 [19, 20, 21].

In recent years, research teams in China have also achieved significant progress on the technological innovations of xenotransplantation [22]. The gene‐editing technology and the production capacity of gene‐edited pigs in China are close to the international leading edge [23]. However, further evidence from preclinical studies in primate models should be presented to support the efficacy and safety of clinical trial of xenotransplantation for human subjects in China.

b. Xenotransplantation also encompasses anthropology, sociology, and ethics, all of which may have significant impacts on our society, hence we must proceed with caution.

The experiences in allogeneic organ transplantation have shown that recipients have the belief that they have acquired qualities or adopted traits from the donors after transplantation [24, 25]. Therefore, human xenotransplantation might bring ethical dilemmas pertaining to personal identity and human dignity, which need to be cautiously evaluated in the process of preparation for clinical trial.

In 2008 and 2018, in Changsha China, the World Health Organization (WHO) and the International Xenotransplantation Association (IXA) developed and updated the WHO regulation for xenotransplantation clinical trials (the Changsha Communiqué) with the participation of the Chinese transplant community [26, 27]. The Changsha Communiqué indicated that the Chinese transplant professionals held the ethical and legal issues associated with xenotransplantation in high regard. In 2009 and 2016, the IXA established and updated the consensus statement on the conditions for undertaking clinical trials of porcine islet products in type 1 diabetes, serving as important references for the international community alongside the scientific progress and regulatory framework in xenotransplantation [28, 29]. The ethical principles and legal consideration in these references provided an important international perspective for the Chinese transplant community in developing the consensus on the xenotransplant clinical trials with the consideration of the medical, legal, ethical, and social conditions in China.

The construction of policy and regulatory framework on xenotransplantation in China is yet to be refined. As outlined in the Changsha Communiqué, any clinical trial should be done under national regulatory oversight. China, as does other countries, needs to develop a national regulatory framework that will allow for appropriate research and a pathway to clinical trials [30], which include but not limited to (1) animal welfare considerations where the national guidelines for the breeding and housing of trial‐use animals [31] must be strictly followed, and (2) patient inclusion mechanism [32] where criteria for recipient selected for xenotransplant instead of allotransplant should be well defined.

In addition, while the participation of industry partners in research and development should be encouraged, the interference from the profit‐seeking capitals must be avoided to safeguard bioethical principles. Xenotransplantation research must abide by the “Guiding Opinions on Strengthening the Ethical Governance of Science and Technology [33]” of China to implement ethical requirements throughout the entire process of scientific and technological innovations. Meanwhile, although studies in Western countries found a high potential acceptance of xenotransplantation among responder to surveys [34, 35], stakeholder perceptions may differ across cultures. The public opinion on xenotransplantation should be closely monitored to reflect the Chinese people's social attitudes and concerns. It is important to be sensible to the perspective that is unique to the Chinese community while keeping up with the international trends.

c. Human organ donation and transplantation in China has advanced in recent years in terms of legal system developments and technological innovations and is entering a new stage of high‐quality development. The key to further advance transplantation in China is still to promote organ donation and allogenic human organ transplantation. The entire medical community, particularly the organ transplant professionals, should continue to promote and improve the ongoing organ donation and transplantation reform in China to provide high‐quality transplant services to Chinese people. Scientific research in allogeneic and xenogeneic organ transplantation should be both supported, as the efforts to foster the development of high‐quality, ethical and sustainable organ transplantation in China.

## AUTHOR CONTRIBUTIONS

**Jiefu Huang**: conceptualization (lead); writing—original draft (lead).

## CONFLICT OF INTEREST

The members of the COTRS scientific committee who also serve on the advisory board, the editorial board, or the emerging editorial board of Health Care Science were not involved in the peer‐review and the decision‐making process of the present manuscript.

## ETHICS STATEMENT

Not applicable. No patient was involved in the present study.

## INFORMED CONSENT

Not applicable.

## Supporting information

Supporting information.

## Data Availability

Data sharing not applicable to this article as no datasets were generated or analyzed during the current study
