# Supplementary material for: Expert consensus on clinical trials of human xenotransplantation in China
Source: Health Care Sci. 2022 Jul 27;1(1):7–10. doi: 10.1002/hcs2.6 (PMC11080631; doi:10.1002/hcs2.6)
Supplement: Supplementary file 1 — Supporting information. [file HCS2-1-7-s001.docx]

**Supplementary material**

Members of the COTRS scientific committee:

*President*

Jiefu Huang

*Vice president*

Yanhong Guo, Haipeng, Xiao, Haibo Wang, Hongtao Zhao

*Secretariat*

Miao Pu, Ying Shi, Jie Zhao

*Members*

Jingyu Chen, Zheng Chen, Zhishui Chen, Zhonghua Chen, Jiahong Dong, Niangguo Dong, Bing Du, Jia Fan, Guiwen Feng, Min Gu, Jianxing He, Xiaoshun He, Shengshou Hu, Feng Huo, Li Li, Tingbo Liang, Lianxin Liu, Yong Liu, Yongfeng Liu, Chung-Mau Lo, Guoyue Lu, Xudong Ma, Tongyi Men, Zhihai Peng, Miao Pu, Haizhi Qi, Zhongyang Shen, Bingyi Shi, Ying Shi, Xuyong Sun, Ye Tian, Ningli Wang, Weilin Wang, Xuehao Wang, Yi Wang, Guosheng Wu, Xiaotong Wu, Qiang Xia, Xiao Xu, Wujun Xue, Hongji Yang, Jiayin Yang, Yang Yang, Qifa Ye, Xiaomei Zhai, Shuijun Zhang, Hong Zheng, Shusen Zheng, Zhe Zheng, Jiangqiao Zhou, Jiye Zhu, Youhua Zhu, Zhijun Zhu.
